# Characterization of Purified Mulberry Leaf Glycoprotein and Its Immunoregulatory Effect on Cyclophosphamide-Treated Mice

**DOI:** 10.3390/foods11142034

**Published:** 2022-07-09

**Authors:** Yangwei Shan, Chongzhen Sun, Jishan Li, Xin Shao, Junfeng Wu, Mengmeng Zhang, Hong Yao, Xiyang Wu

**Affiliations:** 1 Department of Food Science and Engineering, Jinan University, Huangpu Road 601, Guangzhou 510632, China; shanyangwei1115@gmail.com (Y.S.); shaoxincd@163.com (X.S.); wujunfeng953j@163.com (J.W.); 2 School of Public Health, Guangdong Pharmaceutical University, Jianghai Avenue 283, Haizhu District, Guangzhou 510006, China; 3 Faculty of Engineering Technology, KU Leuven, Gebroeders De Smetstraat 1, 9000 Gent, Belgium; jishan.li@student.kuleuven.be; 4 College of Food Sciences and Engineering, South China University of Technology, Guangzhou 510640, China; zhangmengmengscut@126.com; 5 Centre for Nutrition and Food Sciences, Queensland Alliance for Agriculture and Food Innovation, The University of Queensland, St. Lucia, QLD 4072, Australia; hong.yao@uq.edu.au

**Keywords:** mulberry leaf protein, structure analysis, immunosuppression, gut microbiota, correlation analysis

## Abstract

Mulberry leaf protein is a potentially functional food component and health care agent with antioxidant and anti-inflammatory properties. However, its composition, immunoregulatory effects, and gut microbial regulatory effects are unclear. Herein, ultra-filtrated and gel-fractionated mulberry leaf protein (GUMP) was characterized. Its effects on cyclophosphamide-induced immunosuppressed mice were further investigated. The results indicated that GUMP is a glycoprotein mainly containing glucose, arabinose, and mannose with 9.23% total sugar content. Its secondary structure is mainly β-sheet. LC–MS/MS analysis showed that GUMP closely matched with a 16.7 kDa mannose-binding lectin and a 52.7 kDa Rubisco’s large subunit. GUMP intervention significantly improved serous TNF-α, IL-6, and IL-2 contents; increased serum immunoglobulins (IgA and IgG) levels; and reversed splenic damage prominently. Moreover, GUMP administration increased fecal shot-chain fatty acid concentration and up-regulated the relative abundance of *Odoribacter*, which was positively correlated with SCFAs and cytokine contents. Overall, GUMP alleviated immunosuppression through the integrated modulation of the gut microbiota and immune response. Therefore, GUMP could be a promising dietary supplement to help maintain gut health.

## 1. Introduction

Immunosuppression is normally seen in a variety of side-effects of therapies, such as the use of steroids to temper inflammation during COVID-19 and chemotherapy drugs to shrink cancer cells [1,2]. Cyclophosphamide (CP) is a chemotherapeutic agent that has cytotoxicity and can cause immunosuppression during the process of therapy [3,4]. However, severe immunosuppression can increase the risk of fungal and bacterial infection, which is life-threatening for critically ill patients [5,6]. To eliminate immunosuppression after chemotherapies, levamisole hydrochloride (LH) is a broadly used agent, but it also has certain side effects, such as diarrhea and thrombocytopenia [7]. In comparison, nutritional support and herbal medicines are effective and safe approaches against immunosuppression. Ding et al. [8] reported that a polysaccharide derived from the fruits of *Lycium barbarum* was capable of upregulating interleukin-2 (IL-2) and interferon-γ (IFN-γ)) contents. Zhu et al. [9] demonstrated that ovotransferrin enhanced intestine dendritic cell maturation in CP-treated mice [8,9]. Wu et al. [10] also observed that *Hericium erinaceus* polysaccharide protected mice from immunosuppression by increasing peripheral white blood cells and bone marrow nucleated cells [10]. By taking such food supplements, the immune response can be modified, thereby alleviating immunosuppression.

Protein and amino acid supplements are considerable solutions to immunosuppression [11]. A high-protein diet was reported to optimize IL-1 production and phagocytic activity by rat alveolar macrophages [12]. Conversely, insufficient protein intake may result in the impairment of immune response [13]. In addition, increasing evidence indicates that the gut microbiota also plays an important role in regulating immunosuppression [8,14,15,16,17]. Meanwhile, proteins, an important food supplement, can alleviate immunosuppression by altering the composition of the gut microbiota [9,18]. Most previous studies have focused on how polysaccharides mitigate immunosuppression and their regulatory effects on the intestinal microbiota [8,19]. Comparably, how plant-derived proteins regulate the intestinal microbiota associated with improving host immune response remains unclear.

Mulberry trees are widely planted in Asia, and their leaves are used in traditional Asian medicine for their bioactive properties, such as their antioxidative and anti-inflammatory effects. Mulberry leaf extract has thus been proven, in clinical trials, to be effective against diseases, such as type 2 diabetes, by improving glucose tolerance [20]. Furthermore, mulberry leaves are a rich source of protein (17–25% of dry weight) [21]. It has been reported that mulberry leaf protein (MP) has antioxidant activities [22,23]. According to our preliminary studies, MP was found to activate the immune response of RAW264.7 macrophages and significantly increase their pinocytosis capacity (see Figure S1). Therefore, it is worthwhile to further explore its immune activation in vivo. In addition, we employed in vitro fermentation to decipher how MP is used by the gut microbiota. The results illustrated that MP treatment significantly altered the composition of the gut microbiota (see Figure S2A–C), and increased short-chain fatty acid (SCFA) concentration (see Figure S2D, unpublished findings).

However, the impurity and unrevealed structure of MP confine its extensive research and application. Hence, it is necessary to remove its impurities, identify its compositions and structure, and further validate its immunoregulatory effect in vivo. In this study, MP was purified by ultrafiltration and gel filtration. A gel- and ultra-filtrated mulberry leaf protein (GUMP) was consequently obtained. A gradient dose of GUMP was intragastrically administrated to CP-treated mice to investigate its immunoregulatory effect. Changes in the gut microbiota, immune indexes (including cytokine, immunoglobulin, and organ indexes), splenic morphology, and fecal SCFAs were then evaluated. In addition, the correlations between the gut microbiota, immune indexes, and gavage dose were analyzed. This study provides a reference for the rational application of plant leaf protein in clinical immunosuppressive regulation.

## 2. Materials and Methods

### 2.1. Materials and Chemicals

Mulberry leaves (*Morus atropurpurea* Roxb.) were supplied by the Institute of Sericulture and Agricultural Products Processing, Guangdong Academy of Agricultural Sciences (Guangzhou, China) (specimen accession number is CNCP2009) and picked from the Mulberry Planting Base in Baiyun District, Guangzhou, in June 2019. Mulberry leaf protein (MP) was extracted according to the previous methods described by Sun et al. [21,24]. An Amicon^®^ Ultra-15 centrifugal filter, Sephadex^®^ G-150, and CP were purchased from Sigma-Aldrich (St. Louis, MO, USA). Protein marker and Coomassie Brilliant Blue G-250 were obtained from Sangong Co., Ltd. (Shanghai, China). Mouse IL-2 and IL-6, tumor necrosis factor-α (TNF-α), immunoglobulin A (IgA), and immunoglobulin G (IgG) enzyme-linked immunosorbent assay (ELISA) kits were purchased from Neobioscience Technology Co., Ltd. (Shenzhen, China). AR-grade ammonium sulfate, sulfuric acid, NaCl, and NaOH were obtained from Tianjin Damao Chemical Reagent Factory (Tianjin, China). Phenol and Folin phenol reagents were obtained from Maclin Inc. (Shanghai, China). A glycoprotein staining kit was obtained from RealJimes Inc. (Beijing, China).

### 2.2. Ultrafiltration and Column Chromatography

Our previous study showed MP was mainly composed of proteins above 35 kDa (25%), proteins and polypeptides of 6.5 kDa–35 kDa (21%), and some small molecular peptides of 0.5 kDa–6.5 kDa [22]. To retain the main protein composition of MP, we chose the Amicon ^®^ Ultra-15 centrifugal filter with 10 kDa and 30 kDa membranes as a segregator for separating the main proteins from the fractions below 10 kDa and 30 kDa (including small-molecular-weight saccharides and peptides), respectively. The results showed that the protein concentration of the 30 kDa ultrafiltration tube was higher, although the yield was similar (Figure S3). Therefore, we finally chose the 30 kDa ultrafiltration tube for purification (Figure S3). First, MP was dissolved in ultrapure water to obtain the solution at a final concentration of 1 mg/mL. The MP solution was then centrifuged at 5000× *g* for 20 min in the Amicon ^®^ Ultra-15 centrifugal filter. Thereafter, the concentrated retention fraction was collected, freeze-dried, and named UMP. Next, 2 mL of 20 mg/mL UMP was loaded in a chromatography column (ø1.5 cm × 60 cm) filled with Sephadex^®^ G-150. Ultrapure water was used as eluent. The flow rate was controlled at 0.5 mL/min. A280 of retention was measured every 3 min. A sole absorption peak was observed on the “A280–time” curve. Finally, the fraction was collected, freeze-dried, and named GUMP.

### 2.3. Protein, Total Sugar, and Total Phenol Content (TPC) of MP, UMP, and GUMP

The protein concentrations of MP, UMP, and GUMP were determined by a modified BCA assay kit (Sangon Biotech, Shanghai, China). The total sugar contents of MP, UMP, and GUMP were measured according to the Phenol–Sulfuric Acid Assay [25]. The TPC was determined by the Folin phenol colorimetry method [26].

### 2.4. SDS-PAGE and Glycoprotein Staining

The SDS-PAGE method was conducted following the instructions of the SDS-PAGE preparation kit (Sangong Co., Ltd., Shanghai, China). The molecular weight was calculated according to the datasheet of the protein marker. Glycoprotein staining was performed according to a previous study [27]. Horseradish peroxidase and soybean trypsin inhibitor were used as the positive and negative controls, respectively.

### 2.5. Determination of Monosaccharide Composition

The monosaccharide composition was determined by Bo Rui Saccharide Biotechnology Co., Ltd. (Shanghai, China). Briefly, 16 standard monosaccharide samples were prepared as standard stocks (information on these samples was provided in Supplementary Table S2). Then, 10 mg of the sample was weighed precisely in an ampoule, and 10 mL of 3 mol/L trichloroacetic acid was used to hydrolyze the sample for 3 h at 120 °C. Hydrolysate solvent was evaporated under nitrogen, and then 10 mL of water was added for homogenizing. Finally, 100 μL of solvent was mixed with 900 μL of deionized water for centrifugation, and the supernatant was used for Ion Chromatography (Thermo Fisher Scientific, Waltham, MA, USA) analysis. The chromatographic conditions were: chromatographic column: DionexCarbopacTM PA20 (φ3 mm × 150 mm); mobile phase: A: H_2_O; B: 15 mmol/L NaOH; C: 15 mmol/L NaOH and 100 mmol/L NaOAc; flow rate: 0.3 mL/min; injection volume: 5 µL; column oven: 30 °C; detector: electrochemical detector. The gradient conditions used for the separation were: 98.8% A and 1.2% B for 0~18 min; 50% A and 50% B for 20~30 min; 100% C for 30.1~46 min; 100% B for 46.1~50 min; 98.8% A and 1.2% B for 50.1~80 min.

### 2.6. Fourier Transform Infrared Spectroscopy (FTIR)

The FTIR spectra of GUMP were recorded at 25 °C using a Nicolet iS50+iN10 Fourier transform infrared spectrometer (Thermo Fisher Scientific) with a resolution of 8 cm^−1^. GUMP was disposed of, following the description from a previous study [8]. Secondary structure deconvolution was calculated as reported in previous studies [28,29].

### 2.7. LC–MS/MS Analysis

Protein digestion was carried out according to the method of Wiśniewski et al. [30]. The online chromatography separation was performed on the Easy nLC 1200 system (Thermo Fisher Scientific). The trapping and desalting procedure was carried out with a volume of 20 μL of 100% solvent A (0.1% formic acid). Then, an elution gradient of 5–38% solvent B (80% acetonitrile, 0.1% formic acid) in 30 min was used on an analytical column (Acclaim PepMap RSLC, 75 μm × 25 cm C18–2 μm 100 Å). DDA (data-dependent acquisition) mass spectrum techniques were used to acquire tandem MS data on a Thermo Fisher Q Exactive mass spectrometer (Thermo Fisher Scientific) fitted with a Nano Flex ion source. Data were acquired using an ion spray voltage of 1.9 kV and an interface heater temperature of 275 °C. For a full mass spectrometry survey scan, the target value was 3 × 106, and the scan ranged from 350 to 2000 *m*/*z* at a resolution of 70,000 and a maximum injection time of 100 ms. For the MS2 scan, only spectra with a charge state of 2–5 were selected for fragmentation by higher-energy collision dissociation with a normalized collision energy of 28. The MS2 spectra were acquired in the ion trap in rapid mode with an AGC target of 8000 and a maximum injection time of 50 ms. Dynamic exclusion was set for 25 s.

### 2.8. Animals and Experimental Design

Six-week-old male BALB/C mice were purchased from Huafukang Biotechnology Co., Ltd. (Beijing, China) and housed in a 12 h light/dark cycle room under controlled temperature (25 ± 3 °C) and humidity (50 ± 5%). The procedure for care and use of laboratory animals was approved by the Animal Ethics Committee of Jinan University (approval no. IACUC-20210113-11) and complied with all applicable institutions and government regulations regarding the ethical use of the animals. After a 10-day adaption, 8 mice were used as a normal control (NC) group, and the other 40 mice were intraperitoneally injected with 80 mg/kg of CP on days 1, 2, and 3 to induce immunosuppression [8]. Among them, 32 mice were divided into four groups (*n* = 8 for each group), namely MPL, MPM, MPH, and LH, and intragastrically administrated with 30, 90, and 270 mg/kg/d of GUMP and 40 mg/kg/d of LH, respectively, for the next 15 days. The other 8 CP-induced mice were administered drinking water as the model control group (MC). The body weights were measured every two days. After 15-day administration, the mice were sacrificed by cervical dislocation after collecting blood samples. The serum samples were centrifuged and stored at −80 °C. The feces were collected and stored at −80 °C in sterile centrifuge tubes. The thymuses were collected and weighed, and the thymus indexes were calculated as follows:

$$Thymus\;index=\frac{Thymus\;weight\;\left(\mathrm{mg}\right)}{Body\;weight\;\left(g\right)}\;$$

### 2.9. Determination of Cytokines and Immunoglobulins

The levels of IL-2, IL-6, TNF-α, IgA, and IgG in the serum were measured following instructions of the ELISA kits (Neobioscience Technology Co., Ltd., Shenzhen, China).

### 2.10. Histopathological Staining of the Spleen

Paraffin-fixed blocks were serially cut into 5–6 μm-thick coronal sections. For routine histological examination, the paraffin sections were stained with HE. Pictures at 100× and 400× magnifications were taken under a Nikon upright microscope (Nikon Eclipseci Ci-L, Tokyo, Japan).

### 2.11. Transmission Electron Microscopy (TEM) Analysis

Spleen tissues were divided into small pieces (2 mm × 2 mm) and then fixed in 2.5% glutaraldehyde at 4 °C. The samples were cut into ultrathin slices using Ultramicrotome (EM UC7, Leica, Wetzlar, Germany) and observed under a transmission electron microscope (HT7800, Hitachi, Tokyo, Japan).

### 2.12. Determination of Fatty Acids

The fecal SCFAs (acetic acid, propionic acid, butyric acid, and valeric acid) and BCFAs (branched-chain fatty acids: i-butyric acid and i-valeric acid) were measured using GC (gas chromatography). The sample preparation and GC program referred to previous studies [31,32].

### 2.13. Gut Microbiota Analysis

The analysis procedure was adopted from our previous study [32]. Briefly, total bacterial DNA was extracted using the E.Z.N.A.^®^ soil DNA Kit (Omega Bio-tek, Norcross, GA, USA) following the instructions. The primer pair 338F (ACTCCTACGGGAGGCAGCAG) and 806R (GGACTACHVGGGTWTCTAAT) was used to amplify the V3–V4 region of the bacterial 16S ribosomal RNA gene. PCR products were then sequenced using an Illumina Miseq PE300 provided by Majorbio Bio-Pharm Technology Co. Ltd. (Shanghai, China). Operational taxonomic units (OTUs) with 97% similarity were clustered using Usearch (version 7.0) software. Taxonomic information was annotated on the basis of the SILVA Database. Alpha diversity indices were calculated with Mothur (version v.1.30.1, Ann Arbor, MI, USA). Principal coordinate analysis (PCoA) was performed by R software (version 2.15.3, Vienna, Austria) on the basis of weighted UniFrac distances.

### 2.14. Correlation Analysis

Correlation analysis of gut microbes with immune parameters and SCFAs was performed using Spearman’s coefficient based on heatmap analysis (|r| > 0.4).

### 2.15. Statistical Analysis

The data are presented as means ± standard deviation (SD). Significant differences (*p* < 0.05) were evaluated using one-way ANOVA followed by Tukey’s test for multiple comparisons using SPSS (Version 19.0, IBM, Armonk, NY, USA). GraphPad Prism 9.0 (GraphPad Software, San Diego, CA, USA) was used for graph drawing. Origin software was used to deconvolute and fit peaks in the amide I band of GUMP to calculate its secondary structure percentages. PEAKS Studio 8.5 (Bioinformatics Solutions Inc., Waterloo, ON, Canada) was used to analyze the original raw map files collected by mass spectrometry.

## 3. Results and Discussion

### 3.1. Purification, Molecular Weight Distribution, and Chemical Composition of MP, UMP, and GUMP

**Figure 1:**
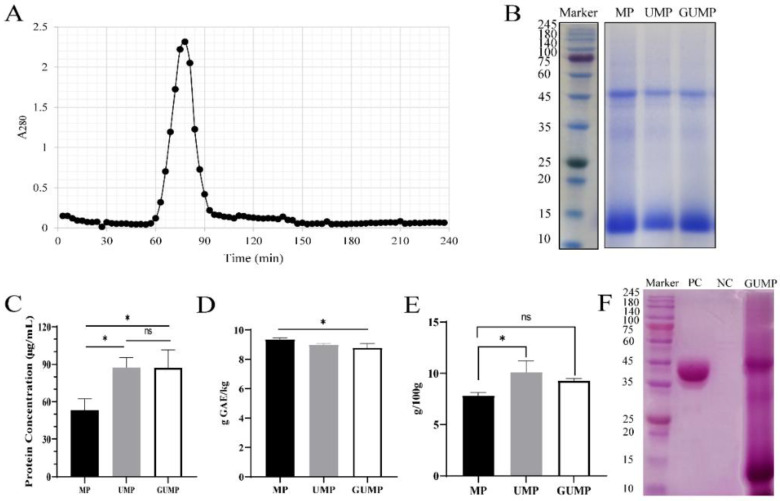
Purification and identification of mulberry leaf protein (MP). Sephadex G-150 chromatography of ultra-filtrated mulberry leaf protein (UMP) (**A**); SDS-PAGE electrophoresis of MP, UMP, and gel-fractionated UMP (GUMP) (**B**); protein concentration determined by BCA kit (**C**); total phenolic contents (**D**); total sugar contents (**E**); glycoprotein staining of GUMP (**F**). PC: positive control, horseradish peroxidase; NC: negative control, soybean trypsin inhibitor. Data are expressed as means ± SD (*n* = 3). * *p* < 0.05.

As shown in Figure 1A, a sole absorption peak was observed, indicating that only one protein fraction was embedded in UMP. Thereby, we collected it as GUMP (gel- and ultra-filtrated MP). As presented by SDS-PAGE electrophoresis (Figure 1B), the protein profiles of MP, UMP, and GUMP were similar, mainly composed of 14 kDa and 52 kDa bands. The molecular masses of MP, UMP, and GUMP were also determined by Native PAGE to be about 100 kDa (Figure S4). After purification, the protein concentrations of UMP and GUMP were 87.67 and 87.33 μg/mL, respectively, which were significantly higher than those of MP (Figure 1C, *p* < 0.05). The result of the TPC analysis is shown in Figure 1D. As expected, the TPC in GUMP was significantly reduced by 0.57 g GAE/kg (*p* < 0.05). Interestingly, as shown in Figure 1E, the sugar contents of UMP (10.12 g/100 g) and GUMP (9.28 g/100 g) were higher than that of MP (*p* < 0.05), implying that our samples might contain saccharides. To validate whether GUMP is a glycoprotein, periodic acid-Schiff staining was then performed [27]. Glycosyls bound to proteins were oxidized to aldehydes by periodic acid. Afterward, the glycosyls were stained, yielding magenta bands with a light pink or colorless background. As shown in Figure 1F, both 52 and 14 kDa subunits were bound with glycosyl, suggesting that GUMP is a glycoprotein. In addition, the protein profile of GUMP was highly similar to that of the 1, 5-ribulose bisphosphate carboxylase/oxidase (Rubisco) present in plant leaves [33]. Researchers have reported that up to 60% of soluble protein in the plant leaf is Rubisco, which is composed of a 50–55 kDa large subunit and a 14–17 kDa small subunit [34,35]. We noticed that some (negligible) bands appeared just below the 52 kDa band. The Rubisco from sugar beet leaves reported by Martin et al. [35] showed a similar pattern. They attributed it to mild proteolysis due to the presence of residual proteases. To further identify whether GUMP is a Rubisco, however, LC–MS/MS should be employed to sequence-specific protein fragments. These results indicate that the purified protein GUMP is a glycoprotein. Our purification method was effective in raising the protein concentration and reducing the TPC in MP, but more experiments are needed to identify GUMP.

### 3.2. Monosaccharide Compositions of GUMP

To further investigate the glycoprotein, ion chromatography was employed to determine GUMP’s monosaccharide. Information on the standard monosaccharide solutions and GUMP are presented in Supplementary Table S2 and Figure S5, respectively. As shown in Table 1, arabinose and glucose were the main compositions in GUMP, and their molar ratios were 2.22 and 2.48 ppm, respectively. In addition, fucose, galactose hydrochloride, rhamnose, glucosamine hydrochloride, galactose, xylose, mannose, and galacturonic acid were detected in the glycosyl of GUMP. Glycoproteins are normally found in plant leaves such as *Azadirachta indica* and *Camellia sinensis* and other food ingredients [36,37,38]. Nie et al. determined the monosaccharide compositions of green tea glycoprotein and found that galactose was the main component, followed by arabinose [37]. Ji et al. [38] also analyzed the monosaccharide compositions in a glycoprotein they prepared from *Salvia miltiorrhiza* and demonstrated that its glycoprotein, SMGP, comprised of rhamnose, arabinose, mannose, glucose, and galactose. Thus, the monosaccharide compositions of glycoprotein may be complex and diverse in plants and foods. Our results further deciphered the structure of GUMP glycoprotein and laid a theoretical foundation for future research.

### 3.3. Characterization of GUMP

In this section, we analyze GUMP’s secondary structure using FTIR and further identified GUMP by LC–MS/MS analysis.

#### 3.3.1. FTIR Analysis

**Figure 2:**
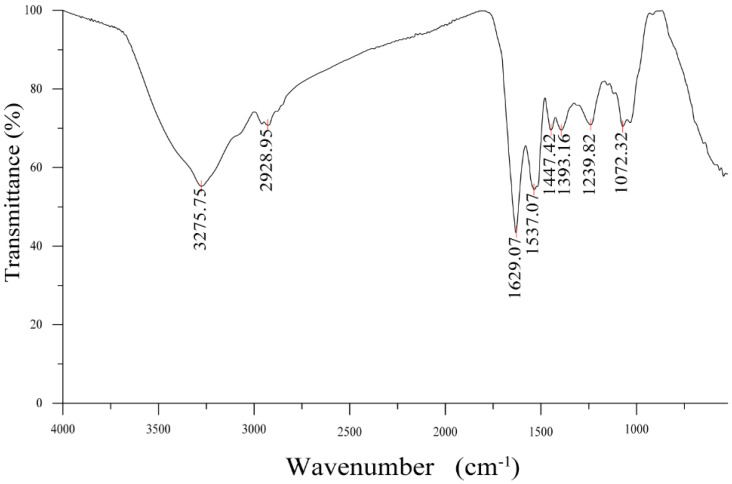
FTIR spectrum of GUMP.

The FTIR result of GUMP is shown in Figure 2, in which the absorption band at 3275.75 cm^−1^ was due to the stretching vibration of O–H vibration, while the weaker shoulder peak at 2928.95 cm^−1^ was attributed to the C–H stretching vibration. The band at 1629.07 cm^−1^ was assigned to the C=C stretching vibration. The characteristic absorption bands of the peptide chain functional group –HN–C=O were 1537 cm^−1^ and 1393 cm^−1^, which were the absorption peaks of the trans configuration and cis configuration of –HN–C=O, respectively.

As shown in Table 2, GUMP was composed of 16.86% α-helix, 46.5% β-sheet, 18.83% β-turn, and 18.58% random coil. According to our previous study, the bands of the β-sheet (1667, 1637, and 1625 cm^−1^) disappeared and the glutamine residues were gradually exposed during the process of simulated gastrointestinal digestion [24].

#### 3.3.2. LC–MS/MS Analysis of GUMP

After matching the sequencing results with the NCBI Morus database, a total of 33 proteins were matched (see Supplementary Table S3), among which the proteins with the highest confidence (364) and the best matching value (81%) was a mannose-binding lectin with a molecular weight of 16.95 kDa (AHW81907.1), followed by a 16.75 kDa protein (AJF21883.1) (see Table 3). In addition, one of the GUMP proteins closely matched with the 52.67 kDa subunit of Rubisco with a confidence level of 155.87. These results confirmed the existence of Rubisco in GUMP but also demonstrated other proteins embedded in GUMP, which was beyond the expectations of this study. However, as the protein information in the NCBI Morus database originated from *Morus alba* var. *atropurpurea*, whereas GUMP was isolated from *Morus atropurpurea* Roxb, it is not guaranteed that the MS/MS matching results are entirely accurate.

Overall, GUMP is a glycoprotein with an 87.33% protein content and a 9.28% sugar content. The most abundant monosaccharides in GUMP were glucose and arabinose, followed by galactose, rhamnose, and mannose. SDS-PAGE showed that GUMP was mainly composed of 14 kDa and 53 kDa bands. Our finding is in agreement with the existence of glycoproteins in the leaves of plants, such as Camellia sinensis [37]. The secondary structure of GUMP was mainly composed of β-sheet (45.65%). The structural evaluation of proteins, especially those naturally occurring in plants, has always been a challenge, as they are complex compounds. In addition, the results of complex protein composition were consistent with the findings reported by Steven J. et al. [39]. Ji et al. and Yun et al. also investigated the nutritional function of glycoproteins isolated from *Salvia miltiorrhiza* and wheat germ, respectively [38,40], but they also failed to reveal how the structure of their samples corresponded to bioactivity as a food supplement. The structure of GUMP is still worthy of further exploration. For instance, the glycosidic bond and the amino acid composition of GUMP should be further investigated in our future studies. In addition, we did not use eluents with strong ionic strengths or organic reagents to fractionate MP because we intended to preserve the protein composition as much as possible and ensure that no salt ions would interfere with our subsequent animal experiments. However, we retained the main proteins of MP while increasing the protein concentration. In this aspect, our fractionation scheme achieved our expectations.

### 3.4. Immunomodulatory Ability of GUMP

#### 3.4.1. Effects of GUMP on the Body Weight and Thymus Indexes in CP-Treated Mice

**Figure 3:**
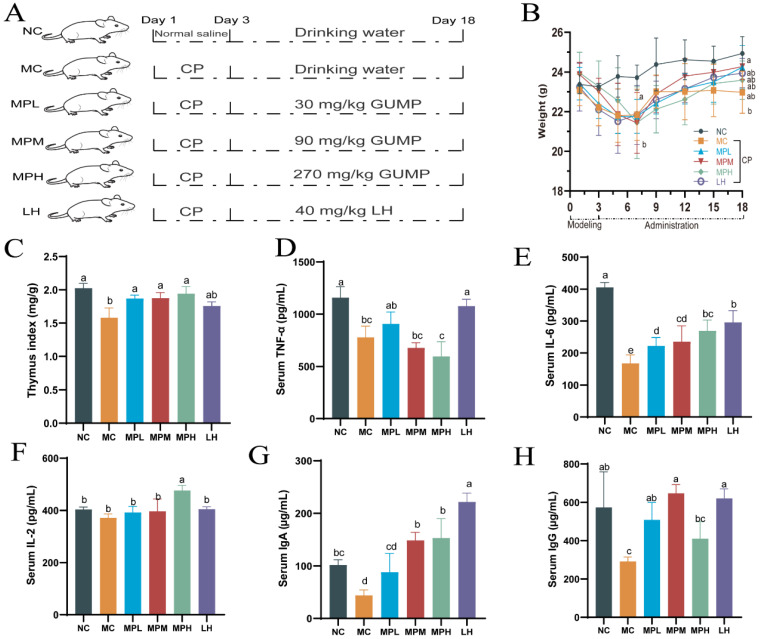
Grouping diagram (**A**); effects of GUMP on the body weight (**B**); thymus indexes (**C**); serous TNF-α (**D**), IL-6 (**E**), IL-2 (**F**), IgA (**G**), and IgG (**H**) of cyclophosphamide-treated mice. Data are expressed as means ± SD (*n* = 8). Different letters represent diverse significant differences, *p* < 0.05.

To evaluate the regulatory effect of GUMP on immunosuppression, a mouse experiment was performed, as shown in Figure 3A. CP intraperitoneal injection can decrease the body weight of mice [8,14]. As shown in Figure 3B, after CP injection for three consecutive days, the bodyweight of CP-induced mice declined. By the seventh day, the bodyweight of CP-induced mice decreased by 5.60% and was significantly lower than that in the NC group (*p* < 0.05). After 7 days, the body weight of mice in the CP-treated group increased gradually, which is consistent with the phenotype observed by Ding et al. [8]. Before the end of the experiment (day 18), the body weight of NC-group mice was significantly higher than that of the MC group (*p* < 0.05). The thymus is an important immune organ in mammals, and its index reflects immune function [41]. CP can induce Treg depletion and thymus impairment, and thus, it decreases the thymus index [42]. Herein, CP significantly reduced the thymus index in the MC group (Figure 3C), while the GUMP treatment reversed the decreased thymus index induced by CP (*p* < 0.05). In addition, there was no significant difference between NC- and GUMP-treated groups (*p* > 0.05). These results indicate that GUMP effectively inhibited weight loss and reversed thymus injury in CP-induced mice.

#### 3.4.2. Effects of GUMP on Serum Cytokine and Immunoglobulin in CP-Treated Mice

A previous study indicated that protein supplements could improve immune function by upregulating cytokine release [43]. To evaluate the effect of GUMP on immunologic function, the levels of IL-2, IL-6, TNF-α, IgA, and IgG in the serum were evaluated. As shown in Figure 3D, the serum TNF-α content in the low-dose GUMP group increased compared with that in the MC group, although there was no significant difference (*p* > 0.05). In addition, the mid- and high-dose GUMP intervention did not recover the TNF-α content. This suggests that only a suitable concentration of GUMP can increase TNF content. IL-6 is an important immune-related cytokine. It is capable of inducing B cells into antibody-producing cells, promoting the growth and differentiation of primitive bone marrow cells, and enhancing the lysis function of natural killer cells [44]. The IL-6 content in GUMP-administered groups was significantly increased compared with that in the MC group (*p* < 0.05) in a dose-dependent manner (Figure 3E). Likewise, Yun et al. reported that wheat germ glycoprotein raised the IL-6 content in intestinal tissue in adult immunosuppressive mice [40]. IL-2 is produced by T cells during immune responses and is critical to naïve T cells and effector T cells [45]. In this study, IL-2 levels in the MPH group were significantly higher than they were in other groups (*p* < 0.001) (Figure 3F). There was no significant difference in the IL-2 level between the MPL, MPM, and MC groups. This observation was also consistent with previous studies [16,46]. Thereby, the increased IL-2 content in the MPH group revealed the stimulative effect on the immunity induced by high-dose GUMP administration. Taken together, the changes in these cytokines suggest that GUMP intervention could alleviate CP-induced immunosuppression by upregulating TNF-α (MPL group), IL-6 (groups MPL, MPM, and MPH), and IL-2 (MPH group) in the serum.

IgG is the main antibody against bacteria, viruses, and toxins in the serum, while IgA is a crucial substance in local anti-infection of the mucous membrane. Both of them play key roles in the immune system [47,48]. In this study, the serum IgA level in the MPM and MPH groups and the IgG level in the MPL and MPM groups were significantly higher than those in the MC group (*p* < 0.05) (Figure 3G,H). In addition, the serum IgA content in the MPL group and the IgG content in the MPH group were higher than those in the MC group, although there was no significant difference (*p* > 0.05). Compared with the recovery effect of ovotransferrin on serous IgA reported by Zhu et al. [9], GUMP raised the content of this indicator, indicating that GUMP alleviated CP-induced immunosuppression and improved host immunity by upregulating serous IgA and IgG levels.

#### 3.4.3. The Effects of GUMP on Histomorphology of Spleen in CP-Treated Mice

**Figure 4:**
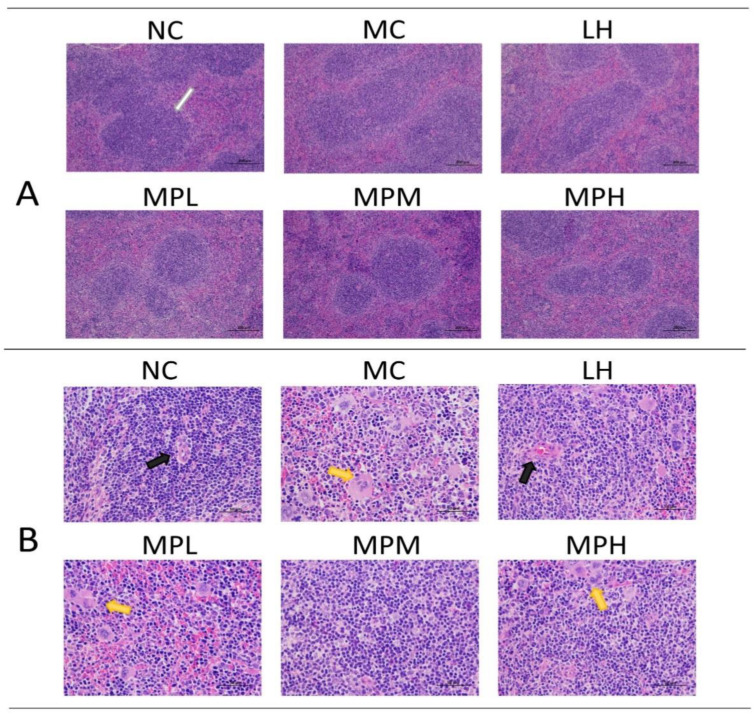
Effects of GUMP on histology of spleen cells (*n* = 5). HE staining of the spleen (×100) (**A**); HE staining of the spleen (×400) (**B**). The white arrow indicates the periarterial lymphatic sheath; the black arrow indicates the central artery; the yellow arrow indicates the megakaryocyte precursor cell.

The spleen is the center of cellular immunity and humoral immunity, accounting for 25% of total body lymphatic tissues, and contains giant eosinophilic cells and lymphocytes. The spleen histology of mice by HE staining is shown in Figure 4A (×100) and Figure 4B (×400), demonstrating that CP caused significant splenic lymphocyte injury and that GUMP administration mitigated this effect. As shown in Figure 4A, the boundary of white and red piths was intact with a compact cell arrangement in the NC group. The CP-induced group showed a blurred boundary of white and red pith, and this change was mitigated by GUMP intervention, particularly in the MPM group. In Figure 4B, megakaryocyte precursor cells emerged. This was mainly due to the reduction in bone marrow hematopoietic stem and progenitor cells (HSPCs) induced by CP [49,50]. Megakaryocyte precursor cells and a loose cell arrangement can be observed in the MC and MPL groups, while the cells were normal and arranged neatly without megakaryocyte precursor cells in the MPM, MPH, and NC groups. These observations indicate that GUMP intervention mitigated CP-induced splenic damage by decreasing the number of megakaryocyte precursor cells and improving the arrangement of splenic cells.

**Figure 5:**
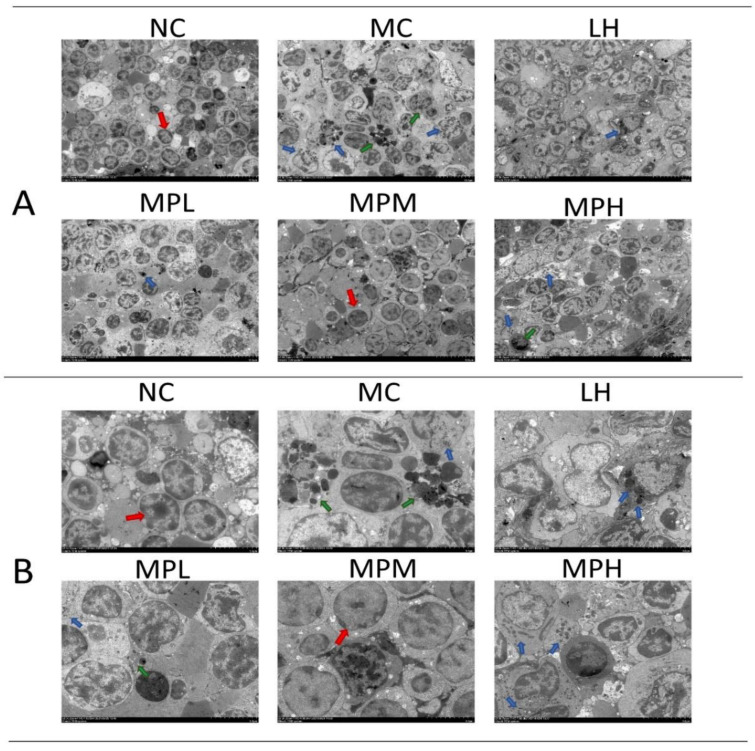
Effects of GUMP on histology of spleen cells (*n* = 5). Transmission electron microscopy of the spleen (TEM; ×1000) (**A**); transmission electron microscopy of the spleen (TEM; ×2500) (**B**). The red arrow indicates the normal splenocytes; the blue arrow indicates the apoptotic bodies; the green arrow indicates the splenocytes undergoing apoptosis.

The submicroscopic and ultrastructural splenocyte arrangement of different groups was observed by TEM (Figure 5A,B). The abnormal shapes of splenocytes in the CP-treated group suggested that the cells were undergoing apoptosis, especially in the MC group, whereas the cells in the MPM group were more regular (Figure 5A). In addition, the apoptosis bodies derived from dead cells could be seen in the CP-treated group (Figure 5A). In the MC group, split cells and distorted cell nuclei were observed (Figure 5B), which could be due to the cell cytotoxicity induced by CP and its metabolites [51]. The decreased persistence of the crosslinks in the G1 phase by CP can lead to lymphocyte death. In addition, CP can inhibit HDAC3 expression, which is essential for the cell cycle, to induce cell apoptosis [52]. The cell shape became regular with fewer visible apoptosis bodies in GUMP-treated groups (Figure 5B), suggesting that GUMP intervention was capable of relieving splenic damage.

#### 3.4.4. Effects of GUMP on SCFA Production in Mouse Feces

**Figure 6:**
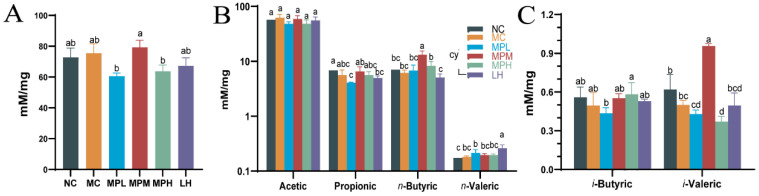
Effects of GUMP on the contents of total fatty acids (**A**), short-chain fatty acids (**B**), and branch-chain fatty acids in feces (**C**). Data are expressed as means ± SD (*n* = 8). Different letters represent diverse significant differences, *p* < 0.05.

Regardless of the digestion of protein and peptides in the small intestine, part of the digested products and amino acids remain to be utilized and fermented by the gut microbiota [53]. SCFAs are metabolites of the gut microbiota, playing a critical role in host metabolism [54], and can regulate the development of immune cells in the intestines and enhance epithelial barrier function by activating inflammasomes [55]. Herein, in comparison with other groups, the total fatty acid content in the MPM group increased to 60.58 mM/mg (Figure 6A), suggesting that mid-dose GUMP administration was better than low- and high-dose administration in promoting SCFA production. Butyrate is a key SCFA that supports the gut lining and can shape immune cell fate [56]. As shown in Figure 6B, the n-butyrate content in the MPM group was significantly higher than that in other groups. Compared with the NC group, the content of n-valeric acid in the MC group was slightly increased. The content of n-valeric acid in the MPL group was significantly higher than that in the NC group (*p* < 0.05), but there was no significant difference compared with the MC, MPM, and MPH groups (*p* > 0.05) (Figure 6B). However, compared with the promoting effect of oyster peptides on SCFAs, GUMP administration was not as effective in promoting acetic acid and propionic acid production [18]. Simultaneously, the i-valeric content in the MPM group remarkably increased compared with that in other groups (*p* < 0.05) (Figure 6C). This was mainly because branched amino acids in proteins and peptides could be used by microbial fermentation to produce branched-chain fatty acids (BCFAs), which are regarded as a marker of protein fermentation [53]. Xiang et al. reported that oyster peptide could significantly restore the level of BCFAs [18]. In this study, the mid-dose GUMP significantly increased i-valeric production compared with other groups (p < 0.05). Collectively, the present results showed that the medium dose of GUMP intervention had the most significant effect on SCFA production and increased the concentrations of n-butyrate and valerate in mouse feces.

#### 3.4.5. The Effects of GUMP on the Gut Microbiota Composition

**Figure 7:**
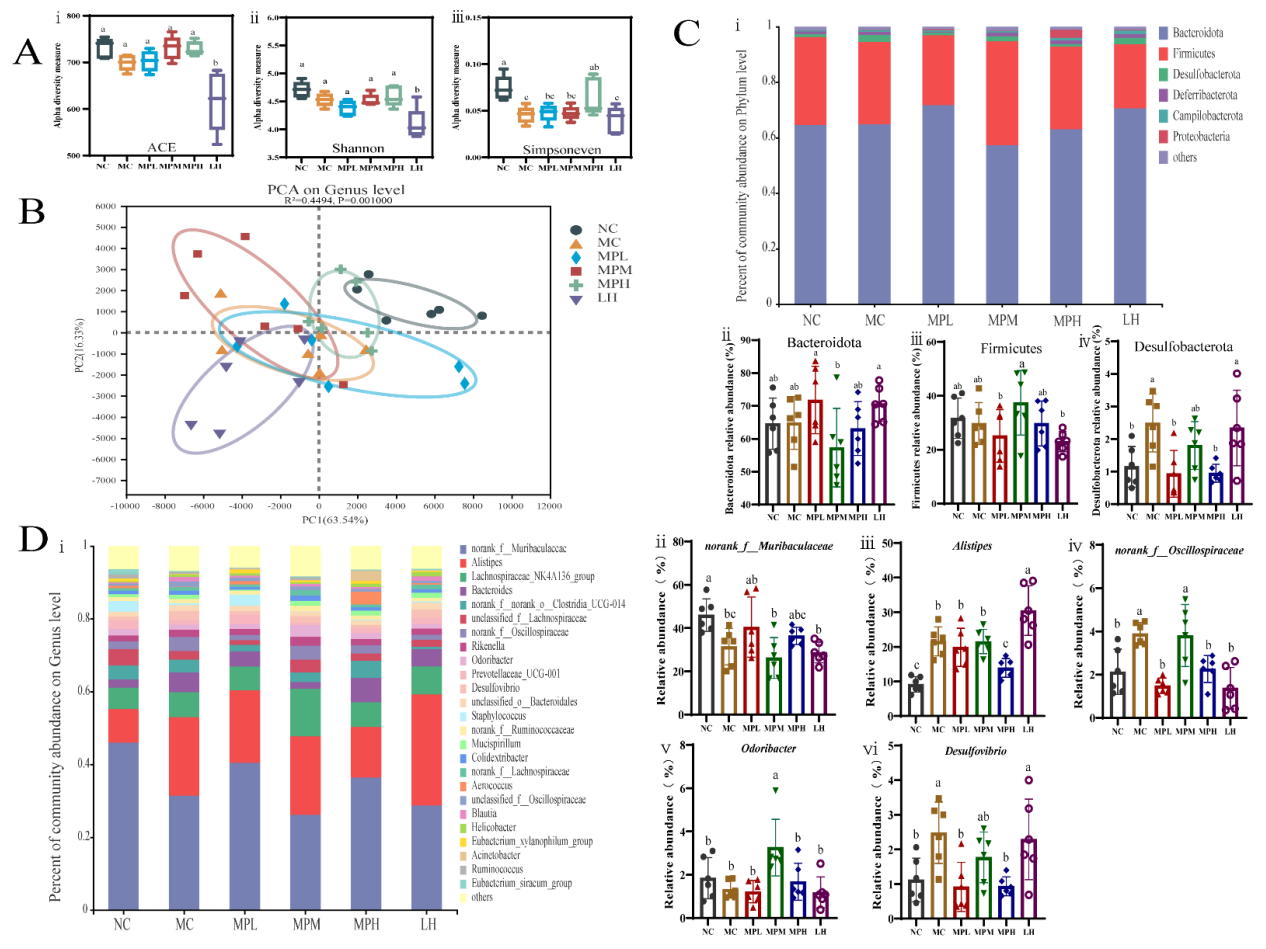
Effect of GUMP on gut microbial composition. (**A**) Alpha diversity estimated through (i) Sobs, (ii) Shannon and (iii) Simpsoneven indexes; (**B**) PCA diagram; (**C**) taxonomic analysis at phylum level: (i) general analysis; (ii–iv): relative abundance of Bacteroidota, Firmicutes and Desulfobacterota, respectively; (**D**) taxonomic analysis at genus level: (i) general analysis; relative abundance of *norank_f__Muribaculaceae* (ii), *Alistipes* (iii), *norank_f__Oscillospiraceae* (iv), *Odoribacter* (v) and *Desulfovibrio* (vi). Data are expressed as means ± SD (*n* = 6). Different letters represent diverse significant differences, *p* < 0.05.

Increasing evidence demonstrates that CP causes intestinal microbiota dysbiosis [57] and that food supplements can alleviate this [19,58]. The moderating effect of GUMP on gut dysbiosis induced by CP was investigated in this study. The 16s rDNA sequence results are depicted in Figure 7.

The alpha diversity was expressed by the ACE, Shannon, and Simpsoneven indexes, as shown in Figure 7A. Compared with the NC group, the MC group showed lower ACE, Shannon, and Simpsoneven indexes (*p* < 0.05). The α-diversity indexes in the MPM and MPH groups were increased compared with that in the MC group and were close to that in the NC group. By contrast, the four indexes in the LH group were significantly lower than in other groups (*p* < 0.05), indicating that the LH group had lower gut microbial diversity. This may be due to the side effects of CP on the gastrointestinal tract and diarrhea caused by LH [41,59]. GUMP intervention also restored the β-diversity. Especially for the MPH group, the grouping ellipse was closer to the NC group (Figure 7B). These results indicated that CP significantly changed the microbial community structure (MC vs. NC), while the GUMP treatment reinstated it. Compared with the β-diversity and low α-diversity indexes caused by LH intervention, protein supplementation exhibited its meliority in alleviating dysbiosis caused by CP.

The taxonomic analysis at the phylum level is shown in Figure 7C. The results illustrated that Bacteroidetes dominated in each group, followed by Firmicutes and Desulfobacterota. As shown in Figure 7C(i,ii), CP injection and GUMP intervention induced no significant alteration in the abundance of Bacteroides and Firmicutes (*p* > 0.05), which was consistent with previous studies [15,60].

However, GUMP intervention reversed the abundance of Desulfobacterota. Firmicutes have been reported to promote the production of butyrate, which might be one of the main reasons for the higher butyrate content in the MPM group (Figure 6B and Figure 7C(ii)) [61]. The relative abundance of Desulfobacterota increased significantly in the MC group compared with the NC group (*p* < 0.05) and decreased in the GUMP-treated groups. Xiang et al. reported that an oyster peptide had a similar effect of ameliorating immunosuppression by reducing the relative abundance of Desulfobacterota, which coincides with our results [18].

To evaluate specific changes in the gut microbiota, the relative abundance at the genus level was then analyzed. As shown in Figure 7D, species with relative abundance higher than 1% were analyzed. Genera potentially related to immune activity were selected and analyzed separately (Figure 7D(ii,vi)). Muribaculaceae was found to be the dominant family in the mouse colon in our previous research [23]. In the present study, the relative abundance of *norank_f__Muribaculaceae* in CP-induced groups was lower than that in the NC group (*p* < 0.05) (Figure 7D(ii)), while low- and high-dose GUMP treatment restored it. It is reported that Muribaculaceae can utilize complex saccharides, and the saccharides bound to GUMP might provide such conditions for *norank_f__Muribaculaceae* growth [62]. *Alistipes* was reported to be potentially pathogenic in colorectal cancer in a previous report [63]. Figure 7D(iii) shows that high-dose GUMP treatment reduced the relative abundance of *Alistipes* significantly (*p* < 0.05) and normalized it to the NC levels. Oscillospira was reported to be positively correlated with inflammation [64]. GUMP treatment could also reverse the relative abundance of *norank_f__Oscillospiraceae* in the MPL and MPH groups to NC level, which was significantly lower than that in the MC group (*p* < 0.05) (Figure 7D(iv)). This implies that GUMP administration might alleviate CP-induced intestinal injury via the reduction in the relative abundance of *norank_f__Oscillospiraceae*. Meanwhile, the genus *Odoribacter*, which was shown to have a positive effect on colorectal cancer [65], was remarkably enhanced in the MPM group compared with other groups (*p* < 0.05) (Figure 7D(v)). *Desulfovibrio* was reported to aggravate inflammation and was positively related to CRC carcinogenesis [66]. GUMP administration also significantly reversed the relative abundance of *Desulfovibrio* in the MPL and MPH groups (*p* < 0.05) (Figure 7D(vi)), revealing that GUMP administration mitigated gut damage by reversing the relative abundance of *Desulfovibrio*. These results illustrate that GUMP administration mitigated CP-induced dysbiosis by upregulating potentially beneficial bacteria, such as *norank_f__Muribaculaceae* and *Odoribacter*, and downregulating potential pathogens, such as *Alistipes*, *norank_f__Oscillospiraceae*, and *Desulfovibrio*. Thereafter, LEfSe analysis was used to explore biomarkers (see Figure S6).

#### 3.4.6. Correlation Analysis of Gavage Dose, Dominant Genera, and Immune Indexes

**Figure 8:**
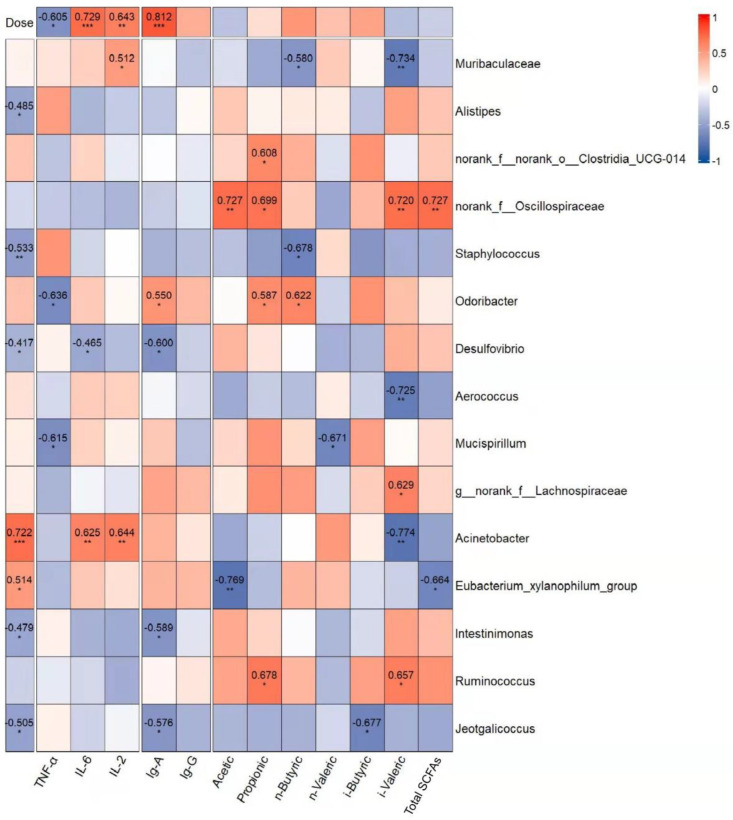
Correlation analysis of gavage dose and the dominant genera (LDA > 2.5 (see Figure S6), relative abundance > 1%) associated with immune indexes: cytokines, immunoglobulins, and SCFAs. The red and blue blocks represent the positive and negative relationships, respectively. The color grade shows the correlation degree. The absolute values of correlation coefficients higher than 0.4 are indicated. * *p* < 0.05, ** *p* < 0.01, *** *p* < 0.001.

To estimate whether the alteration of the intestinal microbiota was related to GUMP administration and immune response, correlations between 15 dominant genera and immune indicators were analyzed. As shown in Figure 8, *Alistipes*, *Staphylococcaceae*, *Desulfovibrio*, and *Jeotgalicoccus* were negatively associated with the dose of GUMP. However, the abundances of *Acinetobacter* and *Eubacterium xylanophilum* were positively associated with the dose of GUMP. *Eubacterium xylanophilum* had a negative relationship with total fatty acids and was considered to have a negative relationship with hepatic GSH and serum HDL-C in a previous study [67]. *Norank_f__Oscillospiraceae* had a positive correlation with multiple SCFA production. In this analysis, *Odoribacter* was the only genus that had a correlative relationship with fecal butyrate. Xing et al. reported that *Odoribacter* is a butyrate producer capable of regulating innate immune signaling [65].

Herein, the increase in the abundance of *Odoribacter* in the MPM group was probably the reason for the high butyrate content. Additionally, the dominant genera *norank_f__Oscillospiraceae* and *Desulfovibrio* in the MC group were negatively correlated with immune indexes (TNF-α, IL-6, IL-2, IgA, and IgG) and gavage dose. These results indicate that GUMP administration could inhibit the species negatively related to immune response. Muribaculaceae, *Eubacterium xylanophilum*, and *Actinobacter* were biomarkers detected in the NC, MPL, and MPH groups, respectively. They had positive correlations with IL-6, IL-2, IgA, IgG, and the gavage dose of GUMP, implying that GUMP intervention enhanced the abundance of certain genera positively related to immune response. Taken together, these genera were correlated with host immune function, and their abundance could be altered by GUMP intervention.

## 4. Conclusions

The qualification of MP was promoted as GUMP after purification. GUMP was identified as a complex glycoprotein that is mainly composed of a β-sheet. Its glycosyl is mainly composed of glucose, arabinose, and galactose. After GUMP administration, the levels of TNF-α, IL-6, IgA, and IgG were significantly increased, and the splenic damage was alleviated. GUMP treatment also stimulated fecal SCFA production, especially for butyrate acid. Furthermore, GUMP regulated certain immune-related microbiota and raised the relative abundance of *Muribaculaceae*, *Odoribacter*, *Eubacterium xylanophilum*, and *Actinobacter*. It reduced the abundance of *norank_f__Oscillospiraceae*, *Alistipes*, and *Desulfovibrio*, which were negatively related to immune response. Collectively, GUMP exhibited an alleviative effect on the immunosuppressed mice, and its ability to restore IL-6 and IgA was higher than that of wheat germ glycoprotein and ovotransferrin reported by previous researchers. Additionally, as a renewable protein source, the recovery effect on the immune response that GUMP exhibited also reflects the prospects of its application. However, some new applications—Alphafold 2, for instance—can still be employed to clarify the structure of GUMP from a new perspective. The mechanism of how gut species mediate immunomodulation still needs to be further investigated.

## Figures and Tables

**Table 1:** The composition of monosaccharides in GUMP.

Monosaccharide	Molar Ratio
Fucose	0.07
Galactosamine hydrochloride	0.02
Rhamnose	0.50
Arabinose	2.22
Glucosamine hydrochloride	0.32
Galactose	0.98
Glucose	2.48
Xylose	0.17
Mannose	0.30
Galacturonic acid	0.25

**Table 2:** The proportions of GUMP’s secondary structure deconvolution.

Secondary Structure	Amide I Band Analysis		
	Characteristic Peaks (cm^−1^)	Peak Area	Percentage Composition
α-helix	1667	16.86	17.4%
β-sheet	1608, 1630	45.65	47.1%
β-turn	1691	18.83	19.4%
Random coil	1648	15.58	16.1%

**Table 3:** Matched protein list of GUMP.

Accession	−10 lgP	Coverage (%)	Peptides	Unique	Spec GUMP	Average Mass	Description
AHW81907.1	364.27	81	51	51	79	16,951	mannose-binding lectin (*Morus alba* var. *atropurpurea*)
AJF21883.1	309.36	75	33	33	45	16,752	18 kD winter accumulating protein (*Morus alba* var. *atropurpurea*)
QVD39028.1	155.87	12	7	6	9	52,668	ribulose-1 5-bisphosphate carboxylase/oxygenase large subunit (chloroplast) (*Morus alba* var. *atropurpurea*)

## Data Availability

Data are contained within the article and the Supplementary Information.

## Supplementary Materials

The following supporting information can be downloaded at: https://www.mdpi.com/article/10.3390/foods11142034/s1, Figure S1: Effects of mulberry leaf protein (MP) and hydrolysates (HMP) on pinocytosis of neutral red (A) and NO production (B). Different letters represent significant differences (*p* < 0.05). MP: mulberry leaf albumin; HMP: neutrase hydrolysates of MP, Figure S2: In vitro fermentation of MP. (A) The ratio of Firmicutes to Bacteroidetes in each group; (B) variation of abundance of *Bacteroidetes*; (C) variation of abundance of *Akkermansia*; (D) variation of organic acids (acetic, propionic, butyric) during fermentation, Figure S3: Effects of 10 kDa and 30 kDa ultrafiltration tubes on protein concentration (A) and sample yield (B), Figure S4: Native PAGE electrophoresis of MP, UMP, and GUMP, Figure S5: Ion chromatographic analysis of standard stock solution (A) and GUMP (B), Figure S6: LEfSe analysis, Supplementary Table S1: Abbreviations and corresponding full names, Supplementary Table S2: Peak information of 16 monosaccharides standards, Supplementary Table S3: Full information of matched protein list of GUMP, Supplementary Table S4: Standard curve of SCFAs.
