# Supplementary material for: Characterization of Purified Mulberry Leaf Glycoprotein and Its Immunoregulatory Effect on Cyclophosphamide-Treated Mice
Source: Foods. 2022 Jul 9;11(14):2034. doi: 10.3390/foods11142034 (PMC9324946; doi:10.3390/foods11142034)
Supplement: Supplementary file 1 [file foods-11-02034-s001.zip › foods-1763462-supplementary.pdf]

Supplementary Files:

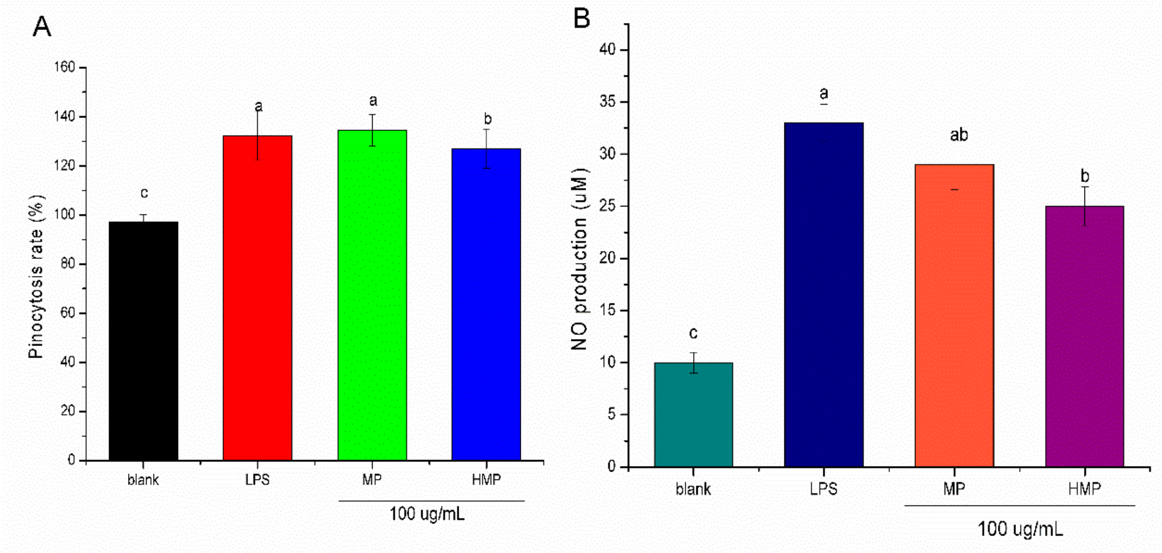

Figure S1. Effects of mulberry leaf protein (MP) and hydrolysates (HMP) on pinocytosis of neutral red (A) and NO production (B). Different letters represent significant differences ($p < 0.05$). MP: mulberry leaf albumin; HMP: hydrolysates of MP. **Results:** Neutral pinocytosis is an important method to detect phagocytosis of macrophages. The stronger the pinocytosis capacity was, the higher the immune activity exhibited. As shown in Fig. S1A, compared with the blank group, the neutral erythron pinocytosis capacity of macrophages was significantly enhanced after MP and HMP were added ($p < 0.05$). Meanwhile, the neutral pinocytosis capacity of MP was significantly higher than that of HMP group. Both MP and HMP could significantly enhance the neutral pinocytosis capacity of RAW264.7 to improve the immune activity, and the effect of MP was better than HMP.

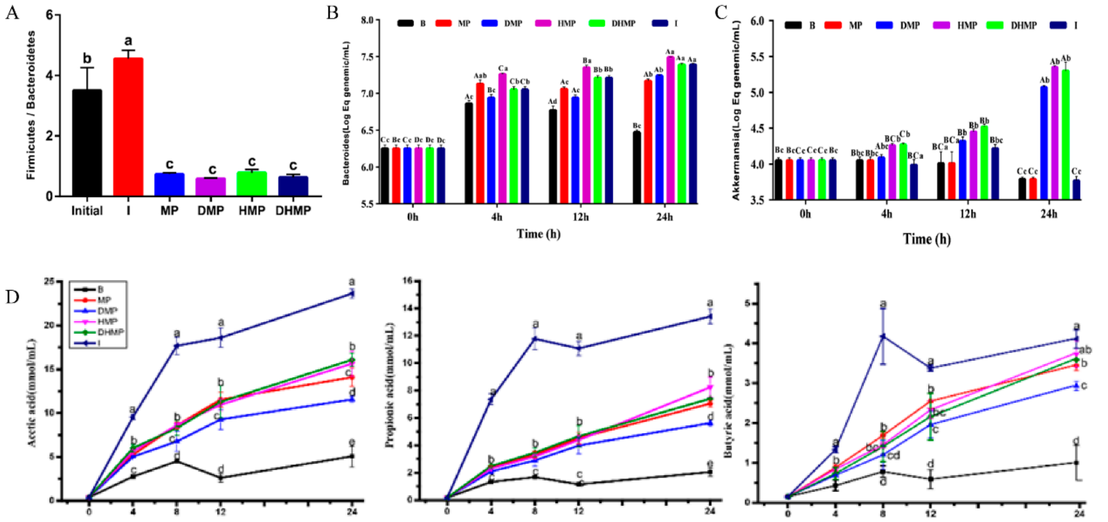

Figure S2. *In-vitro* fermentation of MP. (A) the ratio of Firmicutes to Bacteroidetes in each group; (B) variation of abundance of Bacteroidete; (C) variation of abundance of Akkermansia; (D) variation of organic acids (acetic, propionic, butyric) during fermentation. B: blank; MP: mulberry leaf protein; HMP: hydrolysed mulberry leaf protein; I: inulin. Data were expressed as the mean ± SEM (n = 3). A-D indicated difference for every single sample at different time points and a-e indicated difference of different samples at the same time point. Results: Compared to inulin (positive control), MP and HMP could be better utilized by Bacteroidete and Akkermansia. As shown in Fig. S3(A) and (B), in the middle and late stages of fermentation, the abundance of Bacteroidete and Akkermansia of MP and HMP group were significantly higher than inulin group ($p < 0.05$). However, the amount of organic acid produced by MP and HPM group was lower than that of inulin group ($p < 0.05$). Additionally, both MP and HMP could significantly reduce the ratio of Firmicutes to Bacteroidetes after fermentation.

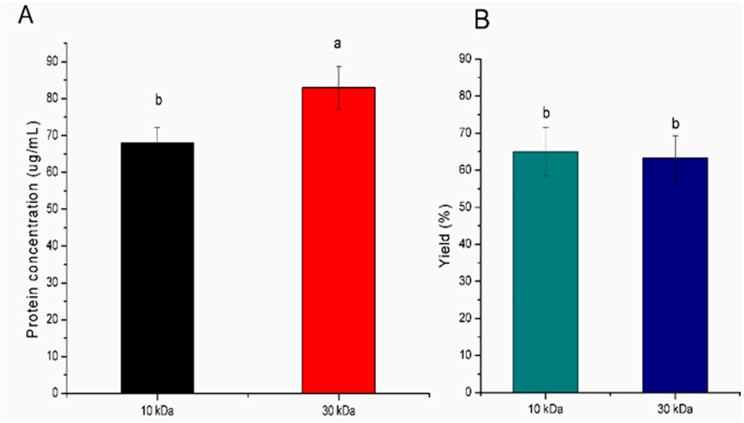

Figure S3. Effects of 10 kDa and 30 kDa ultrafiltration tubes on protein concentration (A) and sample yield (B). Different letters represent diverse significant differences, $p < 0.05$.

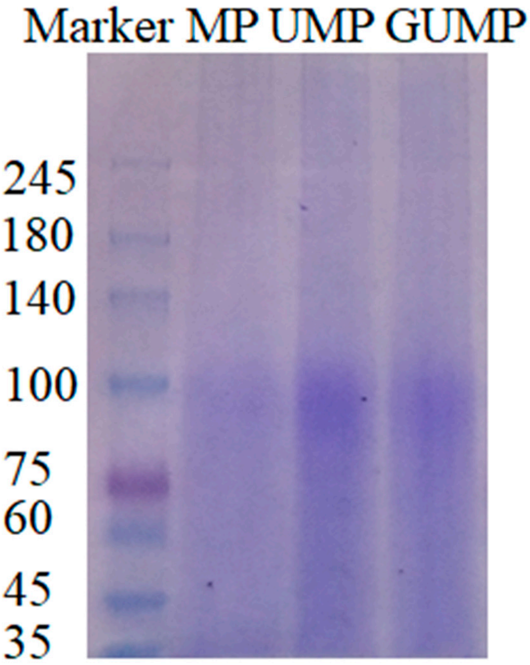

Figure S4. Native-PAGE of MP, ultra-filtrated mulberry leaf protein (UMP) and gel-fractionated UMP (GUMP).

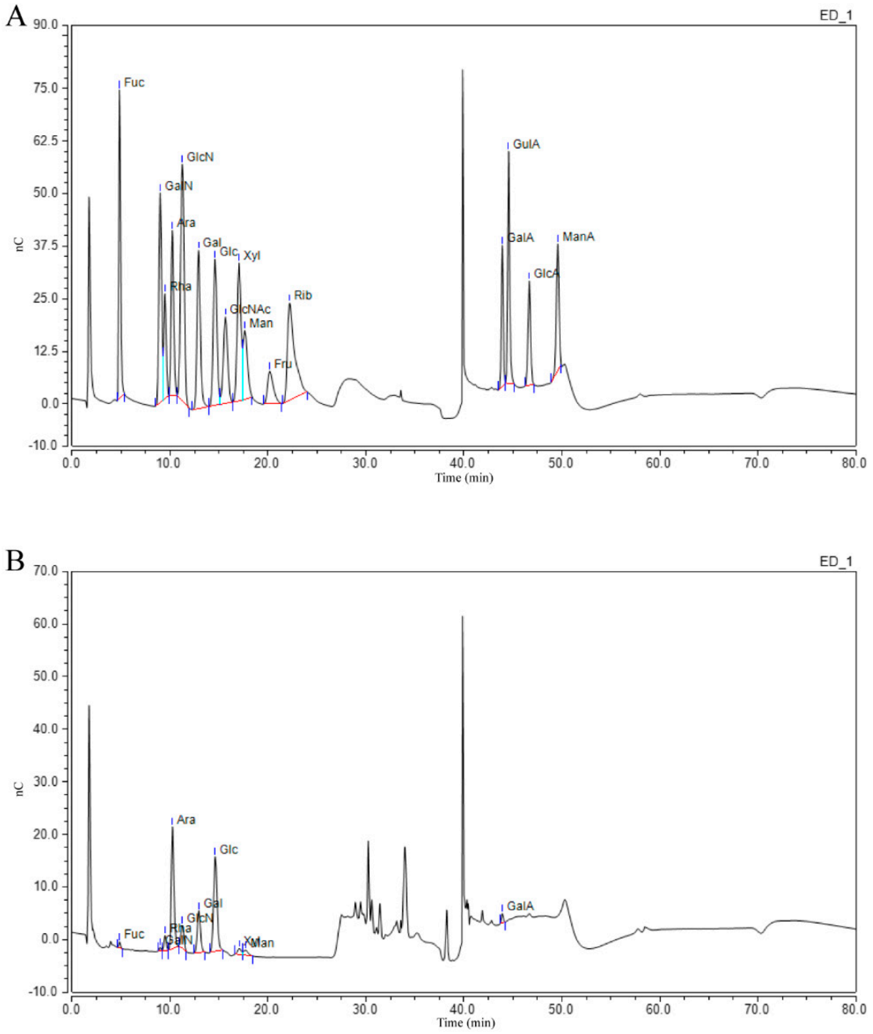

Figure S5. Ion chromatographic analysis of standard stock solution (A) and GUMP (B).

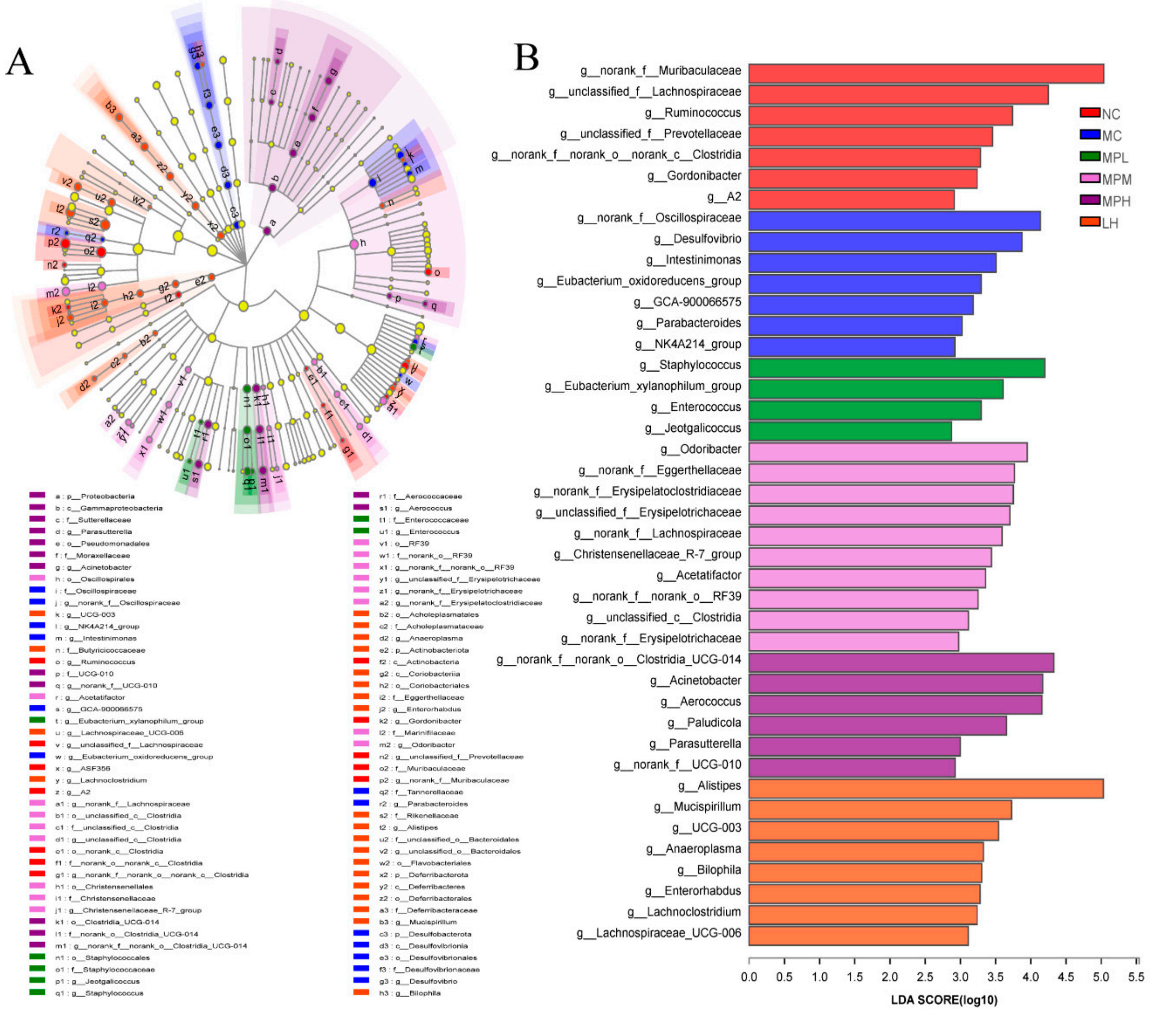

Figure S6. LEfSe analysis. (A) LEfSe results with the threshold value of 2.5 based on a cladogram, whose circles range from phylum to genus; (B) LDA scores from each group with the threshold value of 3 at genus level. As shown in Figure. S6A, all the biomarkers of intestinal microbiota in this study ranging from phylum to genus were exhibited. At the phylum level, Desulfobacterota, Proteobacteria and Actinobacteriota were the biomarkers in MC, MPH and LH groups, respectively. At the order level, Desulfovibrionales showed remarkable prominence in the MC group, and norank_c__Clostridia was the biomarker in the NC group. When it comes to the family level, the Muribaculaceae family was the signature family in the NC group. The Desulfovibrionaceae family showed a striking difference in the MC group; Staphylococcaceae, Marinifilaceae and Clostridia_UCG-014 were the biomarkers in MPL, MPM and MPH group, respectively. Figure S6B displayed the LDA scores at the genus level. Compared with other groups, *norank_f__Muribaculaceae* had the most prominent relative abundance. However, *norank_f__Oscillospiraceae*, which had the highest LDA score, was the biomarker in the MC group, along with the genus of *Desulfovibrio*. The GUMP intervention promoted the relative abundance of *Staphylococcus*, *Odoribacter*, and *norank_f__norank_o__Clostridia_UCG-014*, as biomarkers, in MPL, MPM and MPH groups, respectively. Nevertheless, the most remarkable biomarker in the LH group was *Alistipes*.

Table S1. Abbreviations and corresponding full-name.

Abbreviation	Full name
MP	Mulberry leaf protein
UMP	Ultra-filtrated mulberry leaf protein
GUMP	Gel-fractionated UMP
CP	Cyclophosphamide
GSH	Glutathione
IFN-γ	Interferon-γ
LH	Levamisole Hydrochloride
FTIR	Fourier Transform Infrared Spectroscopy
IC	Ion Chromatography
LC-MS/MS	Liquid Chromatography-Mass Spectrometry
TNF-α	Tumor necrosis factor alpha
IL-1	Interleukin-1
IL-2	Interleukin-2
IL-6	Interleukin-6
IgA	Immunoglobulin-A
IgG	Immunoglobulin-G
GC	Gas chromatography
SCFAs	Short-chain fatty acids
BCFAs	Branch-chain fatty acids
LEfSe	Linear discriminant analysis Effect Size
LDA	Linear Discriminant Analysis

Table S2. Peak information of 16 standards.

Number	Name	Molar ratio (ppm)	Abbreviation	RT (min)	Area
1	Fucose	5	Fuc	4.842	15.395
2	Galactosamine hydrochloride	3	GalN	8.992	16.609
3	Rhamnose	5	Rha	9.475	7.397
4	Arabinose	3.7	Ara	10.225	12.105
5	Glucosamine hydrochloride	5	GlcN	11.250	23.959
6	Galactose	5	Gal	12.909	15.143
7	Glucose	5	Glc	14.584	15.227
8	N-acetyl-D-glucosamine	5	GlcNAc	15.650	10.600
9	Xylose	5	Xyl	17.034	15.282
10	Mannose	5	Man	17.634	8.593
11	Fructose	15	Fru	20.175	5.383
12	Ribose	10	Rib	22.217	19.543
13	Galacturonic acid	5	GalA	43.917	7.306
14	Guluronic acid	10	GulA	44.559	13.091
15	Glucuronic acid	5	GlcA	46.667	7.594
16	Mannuronic acid	10	ManA	49.575	9.542

Supplementary Table S3 Full information of Matched protein list of GUMP

Accession	−10lgP	Coverage (%)	Peptides	Unique	Spec GUMP	Average Mass	Description
AHW81907.1	364.27	81	51	51	79	16951	mannose-binding lectin [Morus alba var. atropurpurea]
AJF21883.1	309.36	75	33	33	45	16752	18kD winter accumulating protein [Morus alba var. atropurpurea]
QVD39028.1	155.87	12	7	6	9	52668	ribulose-1 5-bisphosphate carboxylase/oxygenase large subunit (chloroplast) [Morus alba var. atropurpurea]
ANE10809.1	155.87	12	7	6	9	52668	ribulose 1 5-bisphosphate carboxylase/oxygenase large subunit (chloroplast) [Morus alba var. atropurpurea]
AHW81905.1	140.53	22	6	6	7	18592	pathogenesis-related protein [Morus alba var. atropurpurea]
AHW81904.1	140.53	22	6	6	7	18620	pathogenesis-related protein [Morus alba var. atropurpurea]
AJD79054.1	76.73	26	3	3	3	11372	CPI-3 [Morus alba var. atropurpurea]
ANE10788.1	57.72	2	4	4	4	181961	hypothetical chloroplast RF19 (chloroplast) [Morus alba var. atropurpurea]
QVD39078.1	57.72	1	4	4	4	225464	hypothetical protein RF1 (chloroplast) [Morus alba var. atropurpurea]
AID61550.1	50.76	4	2	2	2	37853	PGIP1 [Morus alba var. atropurpurea]
ANE10815.1	50.63	5	2	2	2	45576	NADH-plastoquinone oxidoreductase subunit 7 (chloroplast) [Morus alba var. atropurpurea]
QVD39076.1	50.63	5	2	2	2	45576	NADH-plastoquinone oxidoreductase subunit 7 (chloroplast) [Morus alba var. atropurpurea]
AKM70873.1	48.15	4	2	2	2	50790	UDP-glucose flavonoid 3-O-glucosytransferase-2 [Morus alba var. atropurpurea]
AJD79055.1	43.43	7	1	1	1	12638	CPI-4 [Morus alba var. atropurpurea]
ANE10791.1	37.4	1	1	1	1	78944	RNA polymerase beta (chloroplast) [Morus alba var. atropurpurea]
QVD39011.1	37.4	1	1	1	1	79829	RNA polymerase beta' subunit (chloroplast) [Morus alba var. atropurpurea]
QVD39053.1	35.4	4	2	2	2	37076	RNA polymerase alpha subunit (chloroplast) [Morus alba var. atropurpurea]
ANE10818.1	35.4	4	2	2	2	37076	RNA polymerase alpha subunit (chloroplast) [Morus alba var. atropurpurea]
ANE10805.1	35.05	1	1	1	1	53734	ATP synthase CF1 beta subunit (chloroplast) [Morus alba var. atropurpurea]
QVD39027.1	35.05	1	1	1	1	53734	ATP synthase CF1 beta subunit (chloroplast) [Morus alba var. atropurpurea]
QVD39004.1	34.7	2	2	1	2	55441	ATP synthase CF1 alpha subunit (chloroplast) [Morus alba var. atropurpurea]

ANE10801.1	34.7	2	2	1	2	55441	ATP synthase CF1 alpha subunit (chloroplast) [Morus alba var. atro-purpurea]
ANE10848.1	32.57	8	1	1	2	8427	hypothetical chloroplast RF68 (chlo-roplast) [Morus alba var. atropur-purea]
ANE10847.1	32.57	8	1	1	2	8427	hypothetical chloroplast RF68 (chlo-roplast) [Morus alba var. atropur-purea]
ANK58711.1	30.54	1	1	1	1	41060	guanine nucleotide-binding protein subunit beta-like 1 protein [Morus alba var. atropurpurea]
ANK58710.1	29.06	1	1	1	1	44643	guanine nucleotide-binding protein subunit alpha-like protein [Morus alba var. atropurpurea]
ANE10794.1	28.92	2	1	1	1	40503	NADH-plastoquinone oxidoreduc-tase subunit 1 (chloroplast) [Morus alba var. atropurpurea]
QVD39075.1	28.92	2	1	1	1	40518	NADH-plastoquinone oxidoreduc-tase subunit 1 (chloroplast) [Morus alba var. atropurpurea]
QVD39024.1	24.71	2	1	1	1	32014	NADH-plastoquinone oxidoreduc-tase subunit K (chloroplast) [Morus alba var. atropurpurea]
ANE10816.1	23.8	2	1	1	2	38893	photosystem II protein D1 (chloro-plast) [Morus alba var. atropurpurea]
QVD38999.1	23.8	2	1	1	2	38893	photosystem II protein D1 (chloro-plast) [Morus alba var. atropurpurea]
QVD39069.1	22.11	2	1	1	1	41092	cytochrome c heme attachment pro-tein (chloroplast) [Morus alba var. atropurpurea]
ANE10819.1	22.11	2	1	1	1	36906	cytochrome c heme attachment pro-tein (chloroplast) [Morus alba var. atropurpurea]

Table S4. Standard curve of SCFAs.

SCFAs	Retention time (min)	Standard curve	R^2
Acetic	3.8	y = 6748.6x - 18774	0.9983
Propionic	4.76	y = 41530x - 1218.5	0.9994
n-Butyric	5.85	y = 107465x + 3150.4	0.9997
n-Valeric	8.12	y = 215471x - 23474	0.9988
i-Butyric	5.09	y = 112808x - 14026	0.9992
i-Valeric	7.27	y = 185054x - 24920	0.9984
